# Cellular metabolism of peripheral CD8+ T cells in early cognitive impairment

**DOI:** 10.1002/alz70861_108845

**Published:** 2025-12-23

**Authors:** Jia Min Tan, Jia Hui Wong, Gurveen Kaur Sandhu, Fatin Zahra Zailan, Jacklyn Leonardo, Xin Ying Sim, Nagaendran Kandiah, Anna M. Barron

**Affiliations:** ^1^ Interdisciplinary Graduate Programme, Nanyang Technological University, Singapore Singapore; ^2^ Lee Kong Chian School of Medicine, Nanyang Technological University, Singapore Singapore; ^3^ Dementia Research Centre (Singapore), Lee Kong Chian School of Medicine, Nanyang Technological University, Singapore Singapore

## Abstract

**Background:**

Interest in the adaptive immune system in AD has been growing recently and it has been shown that CD8+ T cells accumulate in the AD brain, possibly exacerbating neurodegeneration. It is not clear if intrinsic differences in CD8+ T cells in AD patients predispose them to accumulate in the brain. While phenotypic differences in peripheral T cells in AD have been characterised by others, differences in cellular metabolism remains understudied. Given that cellular metabolism can drive immune function, we tested if differences in cellular metabolism can be observed in peripheral T cells in early stages of cognitive impairment.

**Method:**

PBMCs were collected from participants aged 60‐80 in the Biomarker and Cognition Study during their follow up visit. The following groups were examined: Amyloid+ MCI, Amyloid‐ MCI and Amyloid‐ cognitively normal (CN). Amyloid status was determined based on plasma Aβ42:Aβ40 ratio measured on the Simoa platform, which was also used to measure plasma GFAP, NfL, and pTau181. CD8+ T cells were negatively isolated from PBMCs using magnetic cell separation and activated with anti‐CD3 and anti‐CD28 antibodies. Label‐free imaging of cellular NAD(P)H and FAD if hwas used to determine relative levels of glucose catabolism and oxidative phosphorylation. Mitochondria function was assessed using the Seahorse Mitostress assay. Correlation between CD8+ metabolism measures and plasma biomarker concentrations was measured using partial Spearman’s correlation, adjusting for age and sex.

**Result:**

Preliminary results indicate that NAD(P)H levels were significantly higher in Amyloid+ MCI compared to Amyloid‐ CN individuals. A positive correlation between NAD(P)H levels in CD8+ T cells and plasma Aβ42 and Aβ40 concentrations was also observed, whereas the correlation with FAD and plasma GFAP was negative.

**Conclusion:**

Differences in NAD(P)H, a marker of glucose catabolism, can be observed in peripheral T cells in early cognitive impairment. CD8+ cellular metabolism appear to correlate with plasma biomarkers of neurodegeneration and AD, setting the stage for further investigation into the implications on CD8+ function and effect of metabolism‐modifying drugs such as metformin on peripheral CD8+ T cells and AD progression.

